# Health-Related Quality of Life Factors in African American Caregivers of Persons With Dementia

**DOI:** 10.1093/geroni/igaa057.3166

**Published:** 2020-12-16

**Authors:** Glenna Brewster

**Affiliations:** Emory University, Atlanta, Georgia, United States

## Abstract

African American caregivers of persons living with dementia have a high prevalence of caregiving compared to other caregiving groups. The goal of this study was to explore health-related quality of life factors in a sample (n=25) of African American caregivers with a body mass index of more than Caregivers were female (87%). Caregivers were an average age of 63.2 (±6) years, had 16.6 (±2) years of education, and were providing care for 4.3 (±4) years. Care-recipients were on average 79.1 (±11) years and mothers (52.2%). 74% of the caregivers reported good-to-excellent quality of life. Caregivers reported low anxiety and depression but high perceived stress and strain. Higher anxiety and depression were correlated with greater strain and lower quality of life. Healthcare providers need to examine specific situational caregiving factors that can affect African American caregiver outcomes; since, their stress and strain may not reflect in their response to psychological questionnaires.

